# Experience of sexual relationship of patients after radical prostatectomy: a qualitative study

**DOI:** 10.3389/fpsyg.2025.1516544

**Published:** 2025-04-07

**Authors:** Haiyu Zhang, Zhen Liu, Yongmei Ye, Xiaolian Long, Tuan Yu, Yuxi Zhang, Ziling Dai, Juan Du, Ling Chen

**Affiliations:** ^1^The Second Affiliated Hospital of Chengdu Medical College, Nuclear Industry 416 Hospital, Chengdu, Sichuan, China; ^2^Faculty of Humanities and Social Sciences, Macao Polytechnic University, Macao, China

**Keywords:** sexual relationship, radical prostatectomy, qualitative study, prostate cancer, experience

## Abstract

**Background:**

There are different degrees of erectile dysfunction in men after radical prostatectomy, and it takes a long time for their sexual satisfaction to improve. Before the recovery of sexual function, the patient’s sexual relationship experience may change, which may affect the patient’s family stability. Therefore, the present study aimed to comprehensively understand the sexual relationship experience of patients after radical prostatectomy.

**Methods:**

The present study was a qualitative study using the phenomenological method. Purposive sampling was used to select 16 patients with prostate cancer who were followed up after radical prostatectomy in Chengdu, Sichuan Province, and semi-structured interviews were conducted. Colaizzi’s seven-step process for the phenomenological approach was applied to data analysis.

**Results:**

Four themes and 11 subcategories were identified. Four major themes were “differences in the expression of sexual needs,” “changes in sexual behavior,” “changes in intimacy between spouses,” and “changes in role cognition,” respectively.

**Conclusion:**

After radical prostatectomy, many changes occur in the relationship between the sexes. Medical staff should actively guide the relationship between the sexes to help patients and their spouses face up to the changes after surgery, to help improve the relationship between the sexes and improve their quality of life.

## 1 Introduction

As one of the most common malignant tumors in men, Prostate cancer (PCa) has shown an increasing incidence worldwide year by year and has become an important issue affecting men’s health and quality of life (Bray et al., 2018; Kensler and Rebbeck, 2020). Prostate cancer is one of the leading causes of cancer-related death worldwide, with an estimated 1.4 million new prostate cancer cases and 375,000 deaths reported worldwide in 2020 (Adamaki and Zoumpourlis, 2021; World Health Organization, 2021). With the continuous advancement of medical technology, the early diagnosis rate of prostate cancer and the success rate of radical prostatectomy (RP) have significantly improved, and radical prostatectomy has become an important means to treat prostate cancer.

Radical prostatectomy for prostate cancer has achieved remarkable results in improving the survival rate prolonging the survival period of patients, and effectively reducing the incidence of metastasis, but its impact on male sexual function cannot be ignored (Mungovan et al., 2021). Radical prostatectomy can cause damage to the nerves related to sexual function. Postoperative erectile dysfunction, decreased libido, loss of sexual pleasure, and other sexual dysfunction problems are common among patients who have undergone radical prostatectomy (Hoffman et al., 2020). According to a survey, 78 to 87% of patients have erectile dysfunction after radical prostatectomy (Resnick et al., 2013). Although there have been improved surgical methods in recent years that can reduce the degree of injury, the recovery of sexual function in such patients after surgery still takes a relatively long time. In some cases, some patients remain in a state of sexual dysfunction throughout the follow-up period (Agochukwu-Mmonu et al., 2022). Studies have found that the recovery rate of patients’ sexual function score fluctuates between 0 and 40% at 12 months after surgery, and only 3 to 44% at 24 months follow-up (Otaola-Arca et al., 2021). Sexual function problems not only affect patients’ quality of life but also may further affect their mental health, family relationships, and social functioning (Træen and Villar, 2020). Prostate cancer is a family disease, resulting in patients and spouses having a close relationship in decision-making, communication, psychological distress, and other aspects, and significantly affects the relationship and intimacy experience between the couple (Cincidda et al., 2024). Therefore, an in-depth understanding of the sexual relationship experience of prostate cancer patients after radical prostatectomy is of great significance for developing personalized care measures, promoting sexual rehabilitation of patients, and improving the quality of life.

However, due to the influence of traditional Chinese culture and the fact that most prostate cancer patients are older, their sexual relationship experience and sexual health are often overlooked. Sexual health and relationships are an integral part of older people’s general satisfaction with life and well-being, and an important part of healthy aging (Træen and Villar, 2020; Mohamad Al-Ali et al., 2017). Compared with China, other country researchers have conducted certain studies on the sexual health of prostate cancer patients after radical prostatectomy, mostly focusing on the incidence of sexual dysfunction, influencing factors, and intervention measures (Schauer et al., 2015; Chen et al., 2023; Palmer et al., 2018). These studies all adopt quantitative methods and collect data through questionnaires, scale evaluations, etc., which makes it difficult to fully and deeply reveal the true feelings and experiences of patients. As a commonly used research method in the field of sociology, qualitative research can more directly understand the inner world of patients and dig out the meaning and value behind it through in-depth interviews and observations (McGoran et al., 2019; Mottet et al., 2021).

Therefore, the qualitative research method was adopted in this study to systematically explore the sexual relationship experience of prostate cancer patients after radical prostatectomy and understand the changes in postoperative sexual relationship, to provide a more comprehensive and in-depth perspective and ideas for clinical work, and to provide more comprehensive and effective support and help for prostate cancer patients and their families. The hypotheses of this study are as follows: (H1) There are significant changes in the sexual relationship experience of patients after radical prostatectomy; (H2) Changes in sexual relationship experience are complex and heterogeneous; (H3) The intimacy between the patients and their spouses also changed after radical prostatectomy, which affected the relationship pattern between the couple.

## 2 Sample and method

### 2.1 Sample

The purposive sampling method was used to select patients with prostate cancer after radical prostatectomy who were followed up from August 2023 to April 2024 in Grade III and Class A hospitals in Chengdu, Sichuan Province as the study objects. Inclusion criteria: ① The relevant diagnostic criteria in the 2020 European Society of Urology Guidelines for PCa management were met and confirmed by pathological biopsy (Im et al., 2023); ②PCa clinical stage < T4, no distant metastasis; ③radical prostatectomy; ④Age < 80 years old; ⑤Postoperative time ≥ 3 months; ⑥Clear consciousness, normal expression.

Exclusion criteria: ①Patients with serious heart, brain, liver, kidney complications or mental diseases; ②The general condition is poor, and the condition is critical and life-threatening; ③Divorced, widowed or separated from spouse for a long time; ④Cognitive impairment. The sample size of the study is based on information saturation, that is, after the interview to a certain patient no new topics, continue with interview 2 to 3 patients, and still no new topics, it is judged as information saturation. Finally, a total of 16 subjects were included.

### 2.2 Methods

#### 2.2.1 Determine the interview outline

The interview outline was preliminarily formulated according to the purpose of the study by consulting relevant literature, and the interview outline was revised in consultation with 2 urological surgeons, 2 urological clinical nursing experts, and 1 nursing graduate student. The purposive sampling method was used to select 3 patients with prostate cancer after radical prostatectomy for pre-interview, focusing on the selection of patients with characteristics such as talkative, cooperative, and open attitudes towards sexual relationship. Refine the interview outline based on patient feedback to avoid leading questions. The final interview outline includes: (1) How did the relationship between you and your partner change after radical prostatectomy? (2) What is the sexual status of you and your partner after radical prostatectomy? (3) How do you feel about your current sexual life? (4) How do you communicate with your partner about sexual life after radical prostatectomy? (5) What concerns do you have about the intimate relationship with your partner after radical prostatectomy?

#### 2.2.2 Data collection method

Conduct face-to-face interviews with interviewees according to the interview outline. Before the interview, the researcher established a trusting relationship with the interviewees, introduced the purpose and significance of the research to them, and obtained informed consent. Conduct one-on-one interviews with the interviewees in a separate room to ensure that the environment is quiet and comfortable and not disturbed by other unrelated people to protect the privacy of the interviewees. During the interview, a recording device was used for recording. One person interviewed while the other took notes on the spot to record non-verbal materials such as facial expressions and body movements of the interviewees. The duration of each interview is limited to 30 to 60 min.

#### 2.2.3 Data analysis method

Nvivo11.0 software was used to analyze interview data. Transcripts were made within 24 h after the interview, and the transcripts were supplemented and improved based on the notes taken during the interview. Colaizzi’s seven-step Method in phenomenological research was adopted to analyze the data (Bravi et al., 2020). (1) Carefully and repeatedly read the interview materials; (2) Identify meaningful statements that are consistent with the study; (3) Encode recurring ideas that make sense; (4) Assemble the encoded views; (5) Construct meaning units, list common concepts and features, and extract themes and theme groups; (6) Cluster the theme and describe it in detail; (7) Return to the interviewee, verify the results, and ensure the authenticity of the content.

## 3 Results

### 3.1 General characteristics of research objects

A total of 16 subjects were included in this study, all of whom were male, ranging from 56 to 73 years old with a median age of 67.5 years old. All subjects were married with children. Nearly half of the study subjects (7, 43.75%) were retired. Detailed general information is given in Table 1.

**Table 1:** Demographic characteristics of the participants.

Code	Year	Marital status	occupation	Educational level	Clinical stage	Postoperative duration(month)
1	72	Be married	retirement	Primary school	T3bN0M0	8
2	56	Be married	Worker	Junior high school	T3bN0M0	6
3	59	Be married	Retirement	Junior high school	T2cN0M0	6
4	71	Be married	Peasant	Primary school	T2cN0M0	7
5	71	Be married	Retirement	Junior high school	T1cN0M0	7
6	68	Be married	Retirement	Junior high school	T2cN0M0	7
7	64	Be married	Freelancer	Junior high school	T3bN0M0	8
8	69	Be married	Retirement	Junior high school	T1bN0M0	7
9	64	Be married	Worker	Junior high school	T3N0M0	5
10	73	Be married	Retirement	Junior high school	T3bN0M0	4
11	70	Be married	Worker	Primary school	T2cN0M0	4
12	62	Be married	Freelancer	Senior high school	T3N0M0	4
13	69	Be married	Worker	Junior high school	T2cN0M0	4
14	66	Be married	Worker	Junior high school	T2N0M0	6
15	66	Be married	Worker	Postgraduate	T3aN0M0	11
16	67	Be married	Retirement	Undergraduate	T2cN0M0	6

### 3.2 Topic 1:differences in the expression of sexual needs

#### 3.2.1 No need, no expression

In patients with prostate cancer after radical prostatectomy, many individuals show overall indifference or no expression of sexual need. They tend to focus more on recovery and physical health than on sexual health. Therefore, they do not take the initiative to mention sex-related topics, even in daily life also avoid involving, forming a “no demand for sexual life, no expression” state.

In this regard, a patient (Code 12) mentioned:

*“I haven't really thought about that side of things since the surgery. Now every day think about how to make the body quickly better, where also care about other things.”*

Another patient (Code 4) mentioned:

*“At my age, I can't even think about things like that. As long as I don't have a recurrence of this disease and can live a few more years, thank God. It's impossible to talk to her (spouse) about these things.”*

#### 3.2.2 Need, but shy to talk

Although some patients still have an inner need for sex, they often choose to keep this need hidden from their hearts because of the physiological changes brought about by surgery, the pressure of social expectations, and the fear of their partner’s reaction. This psychological state reflects shame, self-doubt, and fear of feedback from partners in the face of sexual dysfunction.

One patient (Code 3) said:

*“Sometimes I want to (have sex), but I don't feel ashamed to say so. That aspect of the function (erection) has not recovered, what to think (embarrassed smile).”*

Nother patient (Code14) mentioned:

*“I still want it sometimes, but every time I see her (my spouse) working so hard to take care of me, I don't know how to ask. After all, there are some failures (sexual function), I am afraid that she thinks I am a waste, more afraid that she will leave me.”*

#### 3.2.3 Need, active expression

Fortunately, there are also a small number of patients and their spouses who can face up to the sexual changes brought about by the surgery and discuss their sexual needs and expectations through open and honest communication. This positive way of communication not only helps to relieve the psychological pressure of the patient but also promotes understanding and support between the partners to find a sexual life solution suitable for both parties.

In this regard, one patient (Code15) mentioned:

*“It was difficult for us at first, but my wife was very understanding and said she would like to face it with me. Neither of us ever felt ashamed to have that need. We talk a lot now, not just about that (sex), but more about how to make our relationship closer.”*

### 3.3 Topic 2:changes in sexual behavior

#### 3.3.1 Traditional sexual behavior loss

In the vast majority of patients, problems such as erectile dysfunction, ejaculation disorders, and pain caused by surgery directly affect the patient’s ability to participate in traditional sexual behavior (that is, sexual activity based on genital contact). The lack of traditional sexual behavior also brings troubles to patients and their spouses to a certain extent, affecting the intimacy of sexual relationship.

A patient (Code 2) said:

*“Although I am in my fifties, my spouse and I used to have an intimate life occasionally before I was diagnosed with prostate cancer. After the surgery, I can no longer function as I used to (erect), which is really frustrating. Now, we never engage in that kind of (traditional) sexual activity anymore, and it feels like something is missing between us.”*

Another patient (Code 10) also mentioned:

*“No, never (shaking head firmly). Even before this (before having prostate cancer), we didn't have those things (sexual life) anymore. She (my spouse) doesn't bring it up either, and now I don't have that function (erection) anymore. It feels more like we're just living together as companions.”*

#### 3.3.2 Non-traditional sexual behavior exploration

Faced with the lack of traditional sexual behavior, some patients and their spouses began to explore non-traditional sexual behavior, while adjusting personal sexual expectations and satisfaction standards to maintain and enhance the intimacy of sexual relationship.

(1) New ways of sexual behavior.

Some patients and their spouses actively explore non-traditional sexual behaviors, such as affectionate hugging, kissing, touching, physical contact, etc., in order to meet the needs of both parties through these activities. It not only helps to relieve the psychological pressure of patients but also enhances the sense of intimacy between each other through touch and emotional communication.

Of these, one patient (Code 7) mentioned:

*“We started to try some new ways of interacting, such as massaging each other, taking baths together, hugging, kissing, and other physical contact more than before. Although there is no direct couple life (traditional sex based on genitals), it also helps us maintain intimacy to a certain extent, which makes me feel at ease.”*

(2) Adjusting expectations and satisfaction criteria.

As sexual behavior changes, patients and their partners also try to adjust their sexual expectations and satisfaction standards. They begin to realize that sexual satisfaction comes not only from genital contact and traditional ways of sexual behavior, but more importantly from emotional communication, physical contact, and understanding and support for each other. This adjustment can help patients reduce anxiety about sexual dysfunction and improve their overall quality of life.

In this regard, one patient (Code 16) mentioned:

*"I always felt that only that (traditional) way could satisfy me, but now I understand that true satisfaction comes from the love we have and the care we have for each other. As long as we can be together, even if it is a pat on the shoulder and holding hands, it is happy."*

Another patient (Code15) also mentioned:

*"In fact, I also lost for a while, but seeing her (spouse) so hard to adapt and change, and often search the Internet for relevant knowledge, I am relieved." Now that we are more emotionally connected and physically connected, we feel happier than before."*

### 3.4 Topic 3:changes in intimacy between spouses

#### 3.4.1 Lack of communication, relationship alienation

After radical prostatectomy, patients often face the stress of functional rehabilitation, emotional fluctuations, and worries about future health can also cause patients to fall into a state of self-isolation, resulting in reduced communication with their spouses. At the same time, the spouse may also feel helpless and anxious because of the changes in the patient’s physical condition, and it is difficult for the two sides to find common topics or emotional outlets, resulting in estrangement.

One of the patients (Code 1) said:

*"After the surgery, I always felt as if I was powerless and even tired of speaking. We communicate less and less, and I know I have become silent, but I just can't control myself. Gradually, she (spouse) didn't talk to me much."*

Another patient (Code 11) had the same experience:

*"I haven't talked much since I was diagnosed. A man, with this disease (prostate cancer), how to face her (spouse)? I barely make eye contact with her now, let alone anything else."*

#### 3.4.2 Increased emotional intimacy

Despite the challenges associated with surgery, some patients have experienced increased emotional intimacy with their spouses after a shared ordeal. In the face of the uncertainty of the disease, the patient and spouse cherish each other’s presence more and survive through mutual support and encouragement. They begin to talk more openly about each other’s feelings and needs and work together to find ways to cope with difficult situations, an experience that promotes intimacy.

One patient (Code 16) mentioned:

*"Although this disease (prostate cancer) made me lose a lot of things, I also gained more value, my relationship with her became better." She used to be more self-centered, not so much concerned about what I thought. After the surgery, she started asking questions and paying more attention to my feelings (grinning). We support each other and feel very secure."*

#### 3.4.3 Changes in daily interactions

In order to relieve the pressure brought about by surgery, some patients and their spouses choose to adjust their daily interaction patterns, by participating in more family activities together, increasing common leisure time, and so on to strengthen their connection and companionship. It not only helps to distract the patient’s attention and reduce his excessive attention to the disease but also enhances the emotional communication between the two sides. In the process of engaging together, patients and partners are able to find new pleasures and common topics, thus enhancing the stability and happiness of the relationship.

Of these, one patient (Code 9) said:

*"We've recently started cooking together, going for walks, and occasionally taking trips nearby. We didn't hang out much in the last couple of decades. Ha ha ha (laughs). These activities let us have more common topics and fun, and sometimes she (my spouse) will play with me like when she was younger."*

### 3.5 Topic 4:changes in role cognition

#### 3.5.1 The reorientation of the patient’s role

After radical prostatectomy, the patient is faced with the role transformation process from “healthy person” to “patient” and then to “convalescent person.” The adaptation of patients to this process varies.

(1) Good role adaptation. Some patients are able to face their situation squarely after surgery, accept reality, and reposition their roles within the family and society. Through positive psychological adjustment and social support, they find new life goals and meaning.

One patient (Code 5) mentioned:

*"Right after the surgery, I really couldn't accept that I had become a 'patient' and always felt like a burden. But gradually, I began to try to accept this fact and try to get better. Now, I don't care so much about what other people think, I just hope that I can live a better life and that my loved one won't have to suffer so much."*

(2) Self-denial. The fear of the disease, concerns about the side effects of treatment, low self-esteem about the decline of physical function, and sensitivity to social prejudice make some patients with prostate cancer suffer from stigma, resulting in a reduced sense of self-worth self-doubt, and denial.

One of the patients (Code13) said this:

*"Ever since I found out about it (prostate cancer), I have been unable to accept the fact. I felt like I was different all of a sudden. Every time I think of myself as a cancer patient, I feel like I'm being labeled and people look at me differently."*

Another patient (Code 6) said:

*"… (Silence for a moment) I am a man and I have this disease (prostate cancer) and I am ashamed to say it. They said that this surgery (radical prostatectomy) may preserve that function (sexual function), but I still have trouble urinating. What's the difference between me and a disabled person now? (with teary eyes)."*

#### 3.5.2 Partner role transformation

The role of the partner often changes dramatically as the role of the patient changes. In the process of recovery, partners need to shift from the traditional role of “supporter” to a more active “caregiver” and “emotional support,” taking on more responsibilities such as household chores, caregiving, and providing psychological support.

A patient (Code 9) said:

*"After the surgery, my wife put almost all her energy on me. She used to like playing mahjong, but now she takes care of me most of the time, afraid that I will not recover well, and also helps me deal with a lot of trifles. I really appreciate her and rely on her more than ever."*

Another patient (Code 3) said:

*"Thanks to her (spouse), she is now the breadwinner of our family, she makes all the decisions. The main reason is that she cares about me and worries that I might overthink things, which could affect my physical recovery."*

#### 3.5.3 Common decision making and responsibility share

In the face of life changes after surgery, some patients and their spouses began to work more closely together, making joint decisions about rehabilitation, sexual life, family affairs, and other issues, and sharing responsibilities and obligations reasonably. This change can not only reduce the pressure burden of a single party but also enable both sides to maintain a better mentality and state to face challenges.

One of the patients (Code 8) mentioned:

*"There are a lot of important decisions that we (patients and spouses) make together throughout the treatment process. For example, choose what treatment plan, how to arrange a rehabilitation plan, and so on. We always consult and support each other. I think this kind of shared decision-making makes us more united and stronger."*

Similarly, another patient (P5) also mentioned:

*"I know it's hard for her, but she still tries to understand and support me." Now at home, from cooking to financial management, she and I have business and quantity and make decisions together. It's a really good feeling to be part of something that makes me feel like I'm not worthless, that we're a community."*

## 4 Discussion

Radical prostatectomy, as an important clinical treatment, can effectively prolong the survival of patients, but its effect on postoperative sexual relationship is a complex and multi-dimensional problem. Through qualitative research methods, this study interviewed 16 patients after radical prostatectomy, conducted an in-depth exploration of their sexual relationship experience, and extracted core themes such as differences in the expression of sexual needs, changes in sexual behavior, changes in marital intimacy, and changes in role cognition, providing useful references for shaping healthy sexual relationship for patients after radical prostatectomy.

The results suggest that there are significant differences in the expression of sexual needs in patients with prostate cancer after radical prostatectomy. Studies have found that many men still desire intimacy and sexual activity after experiencing sexual dysfunction, but they may feel anxious and depressed because of concerns about their own sexual abilities (O'Shaughnessy et al., 2013). Therefore, patients often tend to selectively ignore or suppress their sexual needs. This psychological state not only affects their sexual health but also has a negative impact on their partners’ emotional and psychological well-being. Healthcare professionals should pay greater attention to these patients’ sexual needs, psychological needs, and partner situations when assessing and treating them. Couples-based sexual health education and communication skills training can be provided, and structured couples-based education programs can be developed to help patients and partners learn how to break the silence, establish an open dialog mechanism, and face and express their sexual needs. Resources such as conversation guide cards and emotional expression exercise manuals are provided to help patients and their partners practice expressing their needs and concerns step by step and rebuild a healthy relationship (Bond et al., 2019).

The present study also found significant changes in the sexual behavior patterns of patients after radical prostatectomy. As most patients experienced the dilemma of lack of traditional sexual behaviors, some patients tried to explore other intimate behaviors such as touching, kissing, and hugging. Research indicates a significant association between body self-identity and sexual behavior. Beyond genital-based sexual activities, other forms of physical contact can, to some extent, fulfill sexual needs and enhance sexual satisfaction (Winter and Satinsky, 2014). Therefore, the exploration of non-traditional sexual behavior patterns is highly encouraging, as it can effectively address the deficits in patients’ sexual health. However, some patients still experience confusion and distress when confronted with post-operative changes, feeling too embarrassed to explore new alternative approaches. Glina et al. (2014) found that although many male patients are reluctant to take the initiative to mention sexual health-related issues, they still expect doctors to take the initiative to ask and provide help. Therefore, medical staff should have the ability to identify and manage sexual dysfunction, actively discuss sexual health-related issues with patients during follow-up, and provide patients with comprehensive sexual health guidance, such as sexual skills and sexual replacement therapy training, to help them find their own sexual behavior patterns. Meanwhile, healthcare institutions can collaborate with sexual health experts to develop specific guidance programs. These programs can utilize illustrated manuals, video demonstrations, and other tools to instruct patients and their partners in non-genital intimate activities (such as massage and shared bathing). Additionally, incorporating cognitive-behavioral therapy and similar approaches can help both parties adjust their standards for sexual satisfaction.

The change in marital intimacy is also one of the important changes in the sexual relationship of patients after radical prostatectomy. Some patients and their partners experienced a lack of communication and alienation after surgery. This phenomenon not only affects the mental health of the patient but also negatively affects the emotional support of the partner. Research indicates that mutual support and understanding between partners are crucial when facing significant illness (Ahlberg et al., 2015). Medical staff can help couples feel common emotional support, reduce loneliness, and promote communication between them by organizing couple rehabilitation group activities and other ways (Li et al., 2022). For couples with deeper emotional intimacy, medical staff can provide further emotional support and marriage counseling to help them consolidate and deepen the emotional connection between the couple. Moreover, multidisciplinary working models with professional counselors can be established to guide joint counseling between couples, promote empathy and understanding between couples, and encourage them to share experiences and exchange feelings with other similar families to enhance peer support.

The final theme of this study was role cognitive change. Radical prostatectomy not only changes the physiological state of patients but also profoundly affects the role cognition between men and women. Patients face various physical and mental changes after surgery and have to reposition their roles. During this process, some patients adapt well to their new roles, while others struggle with poor role transitions, including issues such as self-denial. The process of role adaptation is influenced not only by individual psychological states but also closely associated with social and medical risk factors. Patients undergoing radical prostatectomy are often in a state of disease uncertainty because they are not clear about the outcome of postoperative sexual function, urinary control ability, and other physical functions, so they often need higher levels of psychological and social support in the process of adapting to their new roles (Pietilä et al., 2018)^.^ At the same time, the partner, who is an important part of the patient’s life, also needs to complete the transition to become a co-decision maker and share responsibility. This shift not only helps empower patients to make decisions but also improves their overall treatment experience and quality of life. Studies have shown that partner involvement plays an important role in treatment decision-making, is able to reduce patients’ decision regret, and improves their health-related quality of life (Stacey et al., 2020). In addition, partner support can help patients better cope with emotional fluctuations and physical discomfort during treatment, thereby promoting a more active recovery process (Wilding et al., 2020). Therefore, Medical staff should encourage the participation of partners when discussing treatment options with patients and provide them with the necessary support and information to face this challenge together (Owens et al., 2021). Role guidance can also be provided to help patients and their partners gradually adapt to the new role positioning, better cope with postoperative challenges, and promote their mutual growth in postoperative life.

One study noted that prostate cancer patients often face sexual dysfunction and intimacy challenges after surgery, and these problems may manifest and cope differently in different generations of patients (O'Shaughnessy et al., 2013). The younger generation may be more inclined to seek psychological support and modern treatments, while the older generation may rely more on traditional coping mechanisms. In this study, patients aged from 56 to 73 years old, whose sexual attitudes may be deeply influenced by traditional social culture, are more inclined to regard sexual relationship as a private topic, and it takes longer to establish a trusting relationship in communication with medical staff and psychological counselors. Younger patients may be more willing to seek support and accept new-age interventions such as digital intervention tools. Such differences suggest that interventions for the sexual relationship of patients undergoing radical prostatectomy should be designed in layers, dynamically assess the family cultural background, and flexibly adjust the intervention rhythm, such as providing a more private consultation environment and more acceptable forms of health education for the elderly, to better meet the needs of patients of different generations (Bossio et al., 2019).

The study also has some limitations. First of all, this study is a single-center study, and the source of research objects is relatively limited, which may cause the interview results to be affected by geographical, economic, and other objective factors. Secondly, the interviewees in this study were limited to patients and their partners were not interviewed, which may lead to certain role bias in the interview results. It is suggested that future studies should conduct further multi-center interviews and include the patient’s partner in the study to gain a deeper and more comprehensive understanding of the relationship experience and changes of patients after radical prostatectomy.

## 5 Conclusion

The influence of radical prostatectomy on the relationship between men and women is multifaceted, involving the expression of sexual needs, the change of sexual behavior, the change of marital intimacy, and the change of role cognition. Through qualitative research methods, this study reveals the complexity and diversity of these influences in depth, which not only provides a certain reference basis for the comprehensive management of patients undergoing radical prostatectomy from a dual perspective in the future but also suggests the necessity of stratified and personalized intervention. Future studies could further explore the impact of generational and cultural factors on patients’ experience of sexual relationship and intervention outcomes, and optimize intervention programs based on multi-center data to comprehensively improve the quality of life of patients, and their families.

## Data Availability

The raw data supporting the conclusions of this article will be made available by the authors, without undue reservation.
